# Inferences about the transmission of Schmallenberg virus within and between farms

**DOI:** 10.1016/j.prevetmed.2014.04.011

**Published:** 2014-10-15

**Authors:** Simon Gubbins, Joanne Turner, Matthew Baylis, Yves van der Stede, Gerdien van Schaik, José Cortiñas Abrahantes, Anthony J. Wilson

**Affiliations:** a The Pirbright Institute, Ash Road, Pirbright, Surrey GU24 0NF, UK; b Department of Epidemiology and Population Health, Institute of Infection and Global Health, University of Liverpool, Leahurst Campus, Chester High Road, Neston, Cheshire CH64 7TE, UK; c Unit of Co-ordination Veterinary Diagnosis-Epidemiology and Risk Assessment, CODA-CERVA, Groeselenberg 99, 1180 Brussels, Belgium; d Department of Virology, Parasitology and Immunology, Faculty of Veterinary Medicine, Ghent University, Salisburylaan 133, 9820 Merelbeke, Belgium; e GD Animal Health, Arnsbergstraat 7, 7413EZ Deventer, The Netherlands; f European Food Safety Authority, Via Carlo Magno 1A, 43126 Parma, Italy

**Keywords:** Epidemiology, SBV, Modelling, Vector-borne disease, Approximate Bayesian computation

## Abstract

In the summer of 2011 Schmallenberg virus (SBV), a *Culicoides*-borne orthobunyavirus, emerged in Germany and The Netherlands and subsequently spread across much of Europe. To draw inferences about the transmission of SBV we have developed two models to describe its spread within and between farms. The within-farm model was fitted to seroprevalence data for cattle and sheep farms in Belgium and The Netherlands, with parameters estimated using approximate Bayesian computation. Despite the short duration of viraemia in cattle and sheep (mean of 3–4 days) the within-farm seroprevalence can reach high levels (mean within-herd seroprevalence >80%), largely because the probability of transmission from host to vector is high (14%) and SBV is able to replicate quickly (0.03 per day-degree) and at relatively low temperatures (threshold for replication: 12.3 °C). Parameter estimates from the within-farm model were then used in a separate between-farm model to simulate the regional spread of SBV. This showed that the rapid spread of SBV at a regional level is primarily a consequence of the high probability of transmission from host to vector and the temperature requirements for virus replication. Our results, obtained for a region of the UK in a typical year with regard to animal movements, indicate that there is no need to invoke additional transmission mechanisms to explain the observed patterns of rapid spread of SBV in Europe. Moreover, the imposition of movement restrictions, even a total movement ban, has little effect on the spread of SBV at this scale.

## 1 Introduction

During the summer of 2011 dairy cattle in Germany and The Netherlands were reported to be affected by an unknown disease causing a short period of clinical signs including fever, diarrhoea and reduced milk production (Hoffmann et al., 2012, Muskens et al., 2012). Subsequent metagenomic analysis identified the causative agent to be a novel orthobunyavirus (Hoffmann et al., 2012), which has since become known as Schmallenberg virus (SBV). From November 2011 onwards malformations in new-born lambs and calves associated with SBV were reported in Germany, The Netherlands, Belgium, France, Luxembourg, Great Britain, Italy and Spain (European Food Safety Authority, 2012a). The detection of SBV RNA in *Culicoides* biting midges (De Regge et al., 2012, Elbers et al., 2013) suggested that, in common with many other bunyaviruses, SBV is a vector-borne disease.

When a new infectious disease emerges there is little or no information available on its epidemiology or transmission dynamics. In this situation it is possible to use other diseases (ideally ones with some relationship to the novel disease) to provide a framework in which to investigate the potential impact of the emerging disease. In the case of SBV several early studies used models parameterised using data on Akabane virus (a related *Culicoides*-borne virus) and bluetongue virus (BTV) (an unrelated, but well-studied *Culicoides*-borne virus) when exploring scenarios for the spread of SBV (European Food Safety Authority, 2012a, European Food Safety Authority, 2012b, Bessell et al., 2013). However, suitable data, notably from seroprevalence surveys (Elbers et al., 2012, Gache et al., 2013, Méroc et al., 2013a, Méroc et al., 2013b, Veldhuis et al., 2013), are now becoming available and allow inferences about the transmission of SBV to be drawn directly.

In this study we used a stochastic compartmental model, whose structure is similar to one previously developed for BTV (Gubbins et al., 2008, Szmaragd et al., 2009), and fit this to data on the seroprevalence of SBV in cattle and sheep farms in Belgium (Méroc et al., 2013a, Méroc et al., 2013b) and The Netherlands (Veldhuis et al., 2013). Parameters in the model were estimated using approximate Bayesian computation (Marjoram et al., 2003, Toni et al., 2009, Sunnaker et al., 2013). This allows us to avoid calculating a computationally unfeasible likelihood function for the model and instead generates distributions of parameters which are consistent with the within-farm seroprevalence data according to a set of predefined goodness-of-fit metrics.

Once the within-farm parameters had been estimated, their consequences for the spread of SBV at a regional level were explored by incorporating them into a separate, between-farm model adapted from one previously used to describe the transmission of BTV (Turner et al., 2012). Sensitivity analyses were then carried out to explore whether parameter estimates for SBV can account for the different rate of regional spread compared to BTV.

## 2 Materials and methods

### 2.1 Transmission of SBV within a farm

#### 2.1.1 Data

To infer epidemiological parameters for SBV we used data on the within-farm seroprevalence for cattle and sheep farms from Belgium (Méroc et al., 2013a, Méroc et al., 2013b) and The Netherlands (Veldhuis et al., 2013). In total, 422 cattle and 82 sheep farms from Belgium and 543 cattle and 342 sheep farms from The Netherlands were included in the analysis. From each data-set we extracted the number of animals (i.e. cattle or sheep) on the farm, the number of animals sampled, the number of positive samples and the NUTS (Nomenclature of Units for Territorial Statistics) level 2 (NUTS2) region for each farm (European Union, 2011). For The Netherlands, the date of sampling was also extracted.

Temperature data were obtained from the European Commission Joint Research Centre MARS Meteorological Database, which provides daily meteorological data spatially interpolated on a 50 km by 50 km grid cell. Specifically, we extracted the daily minimum and daily maximum temperatures for 2011 and computed the midpoint of these for the pixel closest to the centroid of each NUTS2 region for Belgium and The Netherlands to use in the simulations.

#### 2.1.2 Modelling approach

**Fig. 1:**
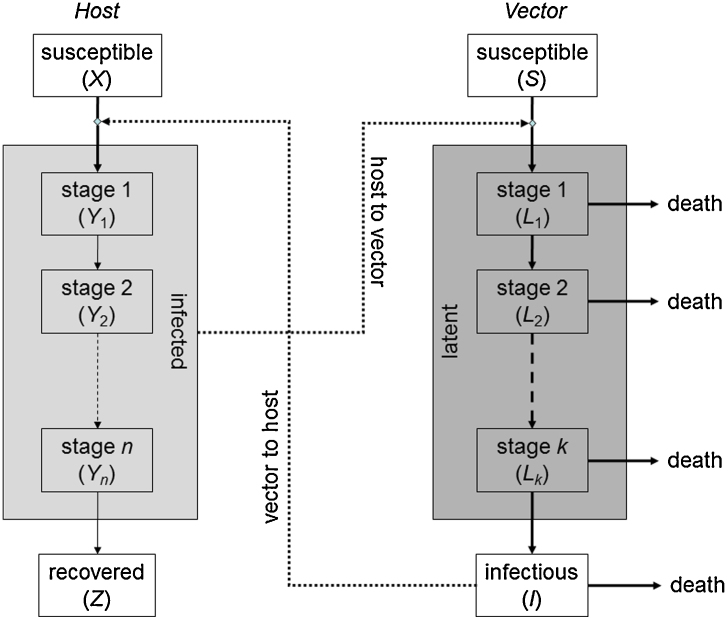
Schematic diagram of the model for the transmission dynamics of Schmallenberg virus within a farm. The populations of infected hosts and latently infected vectors are subdivided into a number of stages to allow for more general distributions for the duration of viraemia and the extrinsic incubation period, respectively. A solid line indicates a flow from one compartment to another; a dotted line indicates that a compartment has an influence on a rate of transfer. Lines shown in bold indicate a temperature-dependent rate.

The within-farm dynamics of SBV were described by a stochastic compartmental model (Fig. 1; Table 1), which was adapted from an earlier model for BTV (Gubbins et al., 2008, Szmaragd et al., 2009). The model includes a single host species (cattle (*C*) or sheep (*S*)), with the total host population (*H*_*i*_) divided into the number of susceptible (*X*^(*i*)^), infected (and infectious) (*Y*^(*i*)^) and recovered (*Z*^(*i*)^) animals (where *i* indicates the species). The duration of viraemia was assumed to follow a gamma distribution with mean 1/*r*_*i*_ and variance 1/*n*_*i*_*r*_*i*_^2^. To incorporate this in the model the infected class (*Y*^(*i*)^) is sub-divided into *n*_*i*_ stages each of mean duration 1/*n*_*i*_*r*_*i*_ (Lloyd, 2001). The vector population (*N*) is divided into the number of susceptible (*S*), latent (i.e. infected, but not yet infectious) (*L*) and infectious (*I*) individuals. To allow for a more realistic gamma distribution for the extrinsic incubation (i.e. latent) period (EIP) (Carpenter et al., 2011), the latent class (*L*) is subdivided into *k* stages each of mean duration 1/*kν* (so the mean duration of the EIP is 1/*ν*). Once infectious, midges remain so for life. Vector mortality occurs at a constant rate *μ* in all classes and is balanced by the recruitment of susceptible vectors, so that the total vector population remains constant.

**Table 1:** Transitions, probabilities and population sizes in the transmission model for SBV.

Description	Transition	Probability	Population size
*Hosts*			
Infection	$\left\{\begin{array}{l} X^{\left(i\right)}\to X^{\left(i\right)}-1\\ Y_{1}^{\left(i\right)}\to Y_{1}^{\left(i\right)}+1 \end{array}\right)$	*λ*_*i*_*δt*	*X*^(*i*)^
Completion of infection stage *j* (*j* = 1,…,*n*_*i*_ − 1)	$\left\{\begin{array}{l} Y_{j}^{\left(i\right)}\to Y_{j}^{\left(i\right)}-1\\ Y_{j+1}^{\left(i\right)}\to Y_{j+1}^{\left(i\right)}+1 \end{array}\right)$	*n*_*i*_*r*_*i*_δ*t*	$Y_{j}^{\left(i\right)}$
Recovery	$\left\{\begin{array}{l} Y_{n_{i}}^{\left(i\right)}\to Y_{n_{i}}^{\left(i\right)}-1\\ Z^{\left(i\right)}\to Z^{\left(i\right)}+1 \end{array}\right)$	*n*_*i*_*r*_*i*_δ*t*	$Y_{n_{i}}^{\left(i\right)}$

*Vectors*			
Infection	$\left\{\begin{array}{l} S\to S-1\\ L_{1}\to L_{1}+1 \end{array}\right)$	*λ*_*V*_*δt*	*S*
Completion of extrinsic incubation period (EIP), stage *j* (*j* = 1,…,*k* − 1)	$\left\{\begin{array}{l} L_{j}\to L_{j}-1\\ L_{j+1}\to L_{j+1}+1 \end{array}\right)$	*kνδt*	*L*_*j*_
Vector mortality (during EIP) (*g* = 1,…,*k*) (and compensatory recruitment)	$\left\{\begin{array}{l} L_{j}\to L_{j}-1\\ S\to S+1 \end{array}\right)$	*μδt*	*L*_*j*_
Completion of EIP	$\left\{\begin{array}{l} L_{k}\to L_{k}-1\\ I\to I+1 \end{array}\right)$	*kνδt*	*L*_*k*_
Vector mortality (infectious vectors) (and compensatory recruitment)	$\left\{\begin{array}{l} I\to I-1\\ S\to S+1 \end{array}\right)$	*μδt*	*I*

The force of infection for host species *i*, *λ*_*i*_, is given by

(1) $$\lambda_{i}(t)=bam_{i}\theta (t)\frac{I(t)}{N}\text{,}$$

where *b* is the probability of transmission from an infected vector to a host, *a* is the reciprocal of the time interval between blood meals for the vector (related to the biting rate), *m*_*i*_(=*N*/*H*_*i*_) is the vector-to-host ratio,

(2) $$\theta (t)\propto \exp\left(b_{0}+\sum_{j=1}^{2}b_{1j}\;\sin\left(\frac{2j\pi t}{365}\right)+b_{2j}\;\cos\left(\frac{2j\pi t}{365}\right)\right)\text{,}$$

is the seasonal vector activity on day *t* (Sanders et al., 2011), normalised so the maximum value is one, and *I*/*N* is the proportion of bites which are from infectious vectors. The force of infection for vectors, *λ*_*V*_, is

(3) $$\lambda_{V}(t)=\beta a\frac{1}{H_{i}}\sum_{j=1}^{n_{i}}Y_{j}^{\left(i\right)}(t)\text{,}$$

where *β* is the probability of transmission from an infected host to a midge.

The basic reproduction number (*R*_0_) for the within-farm transmission model has been derived previously (Gubbins et al., 2008, Gubbins et al., 2012) and can be written explicitly as

(4) $$R_{0}^{\left(i\right)}=\sqrt{\frac{ba}{\mu }{\left(\frac{k\nu }{k\nu +\mu }\right)}^{k}\times \frac{\beta m_{i}a}{r_{i}}}\text{,}$$

where *i* indicates species (i.e. cattle (C) or sheep(S)).

Population sizes in the model take integer values, while transitions between compartments are stochastic processes (Fig. 1; Table 1). The number of transitions of each type during a small time interval *δt* was drawn from a binomial distribution with population size *n* and transition probability *q* (the appropriate *per capita* rate multiplied by δ*t*) (Table 1). However, binomial random variables are computationally expensive to simulate and an approximating distribution was used wherever the following conditions were met. If: (i) *nq*(1 − *q*) > 25; (ii) *nq*(1 − *q*) > 5 and 0.1 < *q* < 0.9; or (iii) min(*nq*,*n*(1 − *q*)) > 10, an approximating normal variate with mean *nq* and variance *nq*(1 − *q*) was used, while if *q* < 0.1 and *nq* < 10, an approximating Poisson variate with mean *nq* was used (Forbes et al., 2011, p. 64).

Parameters in the model are summarised in Table 2. The reciprocal of the time interval between blood meals (related to the biting rate), the vector mortality rate and the mean duration of the EIP were assumed to vary with the local temperature (see Table 2 for details).

**Table 2:** Parameters in the model for the within-farm transmission of Schmallenberg virus (SBV).

Description	Symbol	Prior distribution, estimate or function	Comments
*Inferred using seroprevalence data*			
Probability of transmission from vector to host	*b*	Beta(7.38,2.13)	–
Probability of transmission from host to vector	*β*	Beta(6.60,28.75)	–
Vector to host ratio for species *i*	*m*_*i*_	Triangular(0,1000,5000)^a^	Based on a maximum host biting rate (*m*_*C*_*a*) of 2500 bites per host per day (Gerry et al., 2001); cf. light trap catches of up to 10,000 midges per trap night (Meiswinkel et al., 2008)
Number of animals of species *i*	*H*_*i*_	–	Obtained from the serosurveillance data-sets
Duration of viraemia (cattle)			
Mean (days)	1/*r*_*C*_	Log normal(1.40,0.40)	–
No. stages	*n*_*C*_	Uniform(1,20)	Constrained to take only integer values
Duration of viraemia (sheep)			
Mean (days)	1/*r*_*S*_	Log normal(1.40,0.40)	–
No. stages	*n*_*S*_	Uniform(1,20)	Constrained to take only integer values
Extrinsic incubation period (EIP)			
Mean (days)	1/*ν*	*ν*(*T*) = max(0,*α*(*T* − *T*_min_))	Reciprocal of mean EIP depends on temperature (*T*) (cf. Carpenter et al., 2011)
No. stages	*k*	log normal(2.66,0.77)	Constrained to take only integer values
Virus replication rate above threshold	*α*	Normal(0.019,0.010)	Used to compute reciprocal of mean EIP (1/*ν*)
Threshold temperature for virus replication	*T*_min_	Normal(13.34,1.09)	

*Fixed (not inferred from seroprevalence data)*			
Reciprocal of the time interval between blood meals	*a*	*a*(*T*) = 0.0002*T*(*T* − 3.7)(41.9 − *T*)^1/2.7^	Depends on temperature (*T*) (Mullens et al., 2004)
Vector mortality rate	*μ*	*μ*(*T*) = 0.009exp(0.16*T*)	Depends on temperature (*T*) (Gerry and Mullens, 2000)
Vector recruitment rate	*ρ*	–	For simplicity, assumed to be equal to the vector mortality rate
Vector population size	*N*	–	For simplicity, assumed to be constant; given by *N* = *m*_*i*_*H*_*i*_
Vector activity			
Intercept	*b*_0_	−1.71	Based on analysis of data from a network of 12 suction traps in England (Sanders et al., 2011)
Sin, 12 month period	*b*_11_	−1.56	
Cos, 12 month period	*b*_21_	−3.74	
Sin, 6 month period	*b*_12_	−1.49	
Cos, 6 month period	*b*_22_	−1.00	

a Parameters for the triangular distribution are minimum, mode and maximum.

#### 2.1.3 Approximate Bayesian computation

Nine parameters were estimated by fitting the model to seroprevalence data for cattle and sheep from Belgium and The Netherlands: probability of transmission from vector to host (*b*); probability of transmission from host to vector (*β*); mean duration of viraemia in cattle (1/*r*_*C*_) and sheep (1/*r*_*S*_); number of stages for duration of viraemia in cattle (*n*_*C*_) and sheep (*n*_*S*_); virus replication rate (*α*); threshold temperature for virus replication (*T*_min_); and number of stages for the duration of the EIP (*k*).

Parameters were estimated using approximate Bayesian computation (ABC) rejection sampling (Marjoram et al., 2003, Toni et al., 2009). This allows a joint posterior distribution for the parameters to be generated, but without specifying the full likelihood for the model (which in the case of the model in Section 2.1.2 would be too complex to calculate). In this approach, samples from the posterior distribution are generated as follows:

(a) Sample a parameter set, ***θ***, from the joint prior distribution, *π*(***θ***).
(b) Simulate a data-set, *D*, using the model with the sampled parameter set, ***θ***.
(c) If the simulated data-set, *D*, is sufficiently close to the observed data, *D*_obs_, as judged by an appropriate metric, *M*(*D*,*D*_obs_) (i.e. *M*(*D*,*D*_obs_) < *ɛ*), accept the parameter set; otherwise, reject it.
(d) Return to (a).

Each of the steps (a)–(c) is described in more detail for the SBV model below.

Prior distributions for model parameters were generated using the data from the published literature (Table 2). For some parameters data relating to SBV were available, but, where this was not the case, data for BTV were used instead. This was because there were insufficient data available to construct priors based on other orthobunyaviruses, but also because earlier modelling studies had used estimates based on data for BTV. Specifically:

(i) *Probability of transmission from vector to host* (*b*): a Beta(7.38,2.13) prior was used; this is the posterior derived by Lo Iacono et al. (2013) from an analysis of data on the transmission of BTV to sheep by *Culicoides sonorensis* (Baylis et al., 2008).
(ii) *Probability of transmission from host to vector* (*β*): a Beta(6.60,28.75) prior was constructed such that expected value is equal to the posterior mean and 50% of the prior mass covers the 95% credible interval for *β* derived from data on the experimental infection of *C. sonorensis* with SBV (Veronesi et al., 2013).
(iii) *Mean duration of viraemia in cattle and sheep* (1/*r*_*C*_, 1/*r*_*S*_): log normal priors were used with a mean of approximately 4 days and 95% range of 2–8 days, based on experimental infection of three calves with SBV (Hoffmann et al., 2012).
(iv) *Number of stages for the duration of viraemia in cattle and sheep* (*n*_*C*_, *n*_*S*_): Uniform(1,20) priors were used for these parameters to allow for a range of possibilities from an exponential distribution to an approximately fixed duration of viraemia.
(v) *Virus replication rate* (*α*) *and threshold temperature for virus replication* (*T*_min_): a Normal(0.019,0.01) prior was used for *α* and a Normal(13.34,1.09) prior was used for *T*_min_, where the expected values are equal to the posterior mean and 50% of the prior mass covers 95% of the posterior mass for *α* and *T*_min_ derived from data on the experimental infection of *C. sonorensis* with BTV-9 (Carpenter et al., 2011).
(vi) *Number of stages for the duration of the EIP* (*k*): a prior was constructed by fitting a log normal distribution to the posterior for *k* derived from data on the experimental infection of *C. sonorensis* with BTV-9 (Carpenter et al., 2011).
(vii) *Vector to host ratio* (*m*_*i*_): a triangular prior with minimum zero, mode 1000 and maximum 5000 was used (see Table 2); unlike the other parameters the vector-to-host ratio was sampled independently for each farm to allow for differences amongst farms in terms of farm type (e.g. beef or dairy cattle) or management practices.

All priors were assumed to be independent of one another. The priors were constructed to reflect the relevant data, but without being overly restrictive, thus allowing a wider range of parameter space to be explored.

Once a parameter set had been generated from the priors, the SBV model was run for each farm in the data-set from the time of infection (see below) to the time of sampling, using the reported farm size (i.e. number of cattle or sheep) and 2011 temperature data for the NUTS2 region in which the farm was located. At the end of the simulation, the number of infected animals sampled was simulated using a hypergeometric distribution with farm size, number of infected animals and number of animals sampled as input parameters. Because the sensitivity and specificity of the diagnostic tests used were both very high, their effects on the number of positive samples were not included in the simulations. However, exploratory analysis suggested this is unlikely to have a large impact on the conclusions of the modelling (results not shown).

For each parameter set the time of infection for a farm was sampled uniformly from 1 June 2011 (which was at least 2 weeks before the earliest estimated case in either Belgium or The Netherlands back calculated from cases of arthrogryposis hydranencephaly syndrome in calves and lambs reported to EFSA; Afonso et al., 2014) and the date on which the farm was sampled. Only the range of dates on which farms were sampled was available for Belgium and, hence, all farms were assumed to be sampled on the earliest date in the range (2 January 2012 for cattle and 4 November 2011 for sheep; Méroc et al., 2013a, Méroc et al., 2013b).

Following Conlan et al. (2012), the metric used was a symmetrised version of the Kullback–Leibler distance (Kullback and Leibler, 1951) between the observed and simulated histograms for the within-herd seroprevalence in each species (cattle or sheep) and country (Belgium or The Netherlands). That is,

(5) $$M=\sum_{i,c}\sum_{j}p_{j}^{\left(i,c\right)}\log\left(\frac{p_{j}^{\left(i,c\right)}}{q_{j}^{\left(i,c\right)}}\right)+q_{j}^{\left(i,c\right)}\log\left(\frac{q_{j}^{\left(i,c\right)}}{p_{j}^{\left(i,c\right)}}\right)\text{,}$$

where $p_{j}^{\left(i,c\right)}$ is the proportion of observed seroprevalences in bin *j* for species *i* in country *c* and $q_{j}^{\left(i,c\right)}$ is the proportion of simulated seroprevalences (in a given replicate) in bin *j* for species *i* in country *c*. The histograms for the seroprevalence in the metric, (5), were constructed using bins of 0–5%, 5–10%, …, 95–100%. To ensure the metric, (5), was always defined one was added to every bin of the observed and simulated histograms. Two alternative metrics were considered: sum-of-squares difference between observed and simulated number of seropositive animals; and the Bhattacharyya metric (Aherne et al., 1998) between observed and simulated histograms for within-herd prevalence. Exploratory analysis indicated that neither of these improved the fit of the model or substantially altered the conclusions of the modelling (results not shown).

Parameters were estimated using all four seroprevalence data-sets in a single analysis. However, each data-set was also analysed independently to explore potential differences in estimates and the impact of the size of the data-set on the estimates. For each analysis, sufficient samples were drawn from the priors (and outbreaks simulated) to generate at least 1000 accepted parameter sets, with *ɛ* chosen such that the acceptance rate was around 0.5–1.0%. This number of accepted parameter sets is sufficient to allow robust posterior inferences to be drawn.

### 2.2 Regional-scale transmission of SBV

To explore the transmission of SBV between farms, we adapted a stochastic model for the spread of BTV between farms in Suffolk and Norfolk, two counties in eastern England (Turner et al., 2012). This is an area measuring approximately 100 km × 100 km, containing over 3000 farms. This model is suitable for adaptation to SBV because both SBV and BTV are transmitted by *Culicoides* biting midges to the same hosts, predominantly sheep and cattle. There is no evidence for significant transmission of either virus by other routes (such as vertical or pseudo-vertical) not included in the model.

In the model, farms are divided into susceptible, exposed and infected states. Transmission between farms is assumed to occur by two mechanisms: animal movements and vector dispersal. Transmission via movements is simulated using recorded animal movements, while transmission via vector dispersal is described by a distance kernel. Rather than describe explicitly the dynamics of infection within a farm, a prevalence curve is used to determine how infectious a farm is to neighbouring farms for both transmission routes based on its time since infection.

Prevalence curves were constructed based on the results of the within-farm model (Section 2.1) using the methods described in the electronic Supplementary material. We constructed curves for seven parameter sets that describe SBV, BTV and BTV with individual parameter values set for SBV (see summary in Table 4). This allowed us to explore the relative effect of individual SBV parameters on the overall differences between SBV and BTV. Parameter values for SBV replaced those for BTV as follows: estimated probability of transmission from vector to host (set 2); estimated probability of transmission from host to vector (set 3); estimated duration of viraemia in cattle and sheep (set 5); and estimated relationship between temperature and EIP (set 6). In addition, we ran simulations for BTV with the short, 2 day, incubation period for SBV (Hoffmann et al., 2012) (set 4).

For the purposes of this work, SBV was introduced on day 182 (i.e. 1 July) at the height of the vector season, with an index farm selected at random for each simulation. The infection status of each farm was then tracked for the rest of the year with the cumulative number of cases and mean distance spread from index case recorded at each time step. One hundred replicates of the model were simulated for each parameter set.

Uncertainty in the model outputs for SBV has not yet been quantified. However, sensitivity analysis is presented for the BTV version of the model in Table S4 of Turner et al. (2012).

## 3 Results

### 3.1 Epidemiological parameters for SBV

**Fig. 2:**
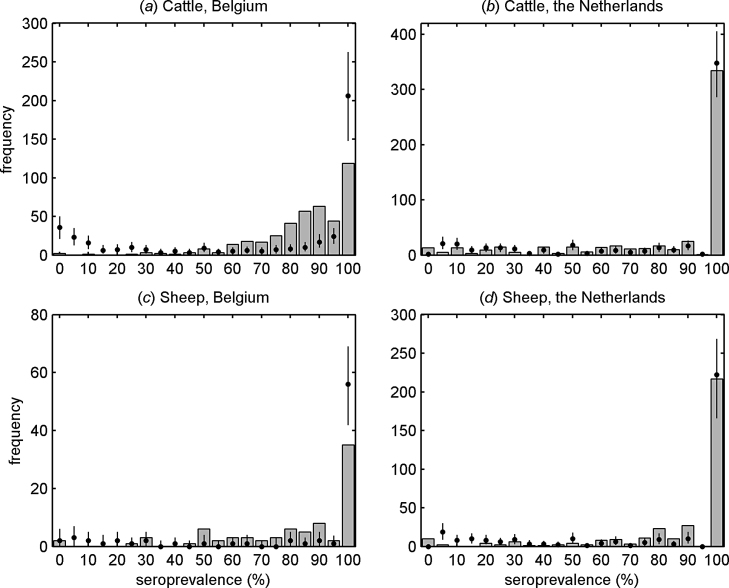
Observed (bars) and expected (median (circles) and 95% prediction intervals (error bars)) distribution of within-farm seroprevalence of Schmallenberg virus in (a, b) cattle and (c, d) sheep from (a, c) Belgium and (b, d) The Netherlands.

The model adequately captures the distribution of within-farm seroprevalence for three out of the four data-sets (cattle in The Netherlands; sheep in Belgium and The Netherlands) (Fig. 2). However, the model results in a poorer fit to the data for cattle in Belgium, overpredicting the frequency of farms with low seroprevalences (0–20%) and underpredicting the frequency of farms with intermediate seroprevalences (60–90%) (Fig. 2).

**Fig. 3:**
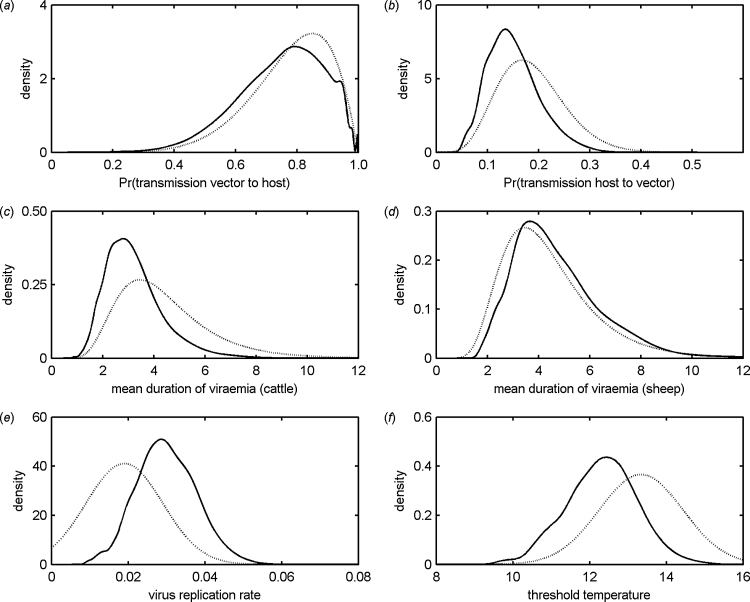
Marginal posterior distributions for epidemiological parameters for Schmallenberg virus (SBV): (a) probability of transmission from vector to host; (b) probability of transmission from host to vector; (c, d) mean duration of viraemia (days) in (c) cattle or (d) sheep; (e) virus replication rate; and (f) threshold temperature (°C) for virus replication. Each figure shows the prior (dotted black line; Table 2) and posterior (solid black line) densities when the model for the within-farm transmission of SBV was fitted to seroprevalence data for cattle and sheep from Belgium and The Netherlands.

The marginal posterior distributions for each parameter are shown in Fig. 3 and summarised in Table 3. Transmission from vector to host was estimated to be very efficient (posterior median for probability of transmission from vector to host: 0.76) (Fig. 3a) and much more so than transmission from host to vector (posterior median for probability of transmission from host to vector: 0.14) (Fig. 3b). The mean duration of viraemia was short in both species, but was estimated to be shorter in cattle (approximately 3 days) than in sheep (approximately 4 days) (Table 3; Fig. 3c and d). The virus replication rate (above the threshold temperature) was estimated to be approximately 0.03 per day-degree (Table 3; Fig. 3e). Finally, the threshold temperature for virus replication was estimated to 12.3 °C (Table 3; Fig. 3f).

**Table 3:** Posterior median and 95% credible intervals (CI) for parameters in the model for the within-farm transmission of Schmallenberg virus (SBV).

Parameter	Median	95% CI
*Probability of transmission*		
Vector to host	0.76	(0.46, 0.95)
Host to vector	0.14	(0.07, 0.26)

*Duration of viraemia (cattle)*		
Mean (days)	3.04	(1.63, 5.91)
No. stages	11	(1, 20)

*Duration of viraemia (sheep)*		
Mean (days)	4.37	(2.24, 9.02)
No. stages	11	(1, 20)

*Extrinsic incubation period*		
Virus replication rate	0.030	(0.016, 0.045)
Threshold temperature	12.35	(10.52, 14.02)
No. stages	6	(2, 35)

Both the vector-to-host ratio and time of infection were allowed to differ amongst farms. The posterior distributions for the vector-to-host ratio did not differ greatly amongst farms or, indeed, from the prior distribution. Posterior estimates for the time since infection suggest that farms could have become infected between late June and early September, but that they were more likely to have been infected between late July and early August.

**Fig. 4:**
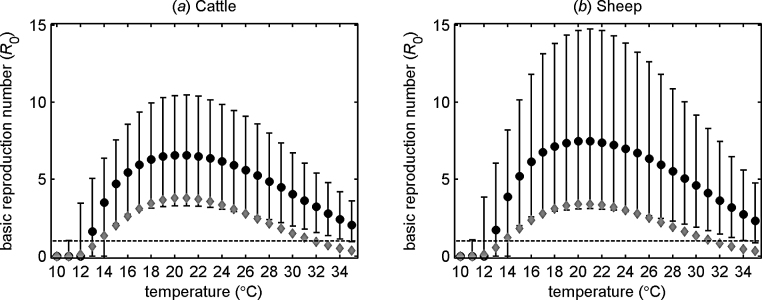
Basic reproduction number (*R*_0_) for Schmallenberg virus in (a) cattle and (b) sheep and its dependence on temperature. Each figure shows the posterior median (circles) and 95% credible intervals (error bars) for *R*_0_ computed using Eq. (4). The black dashed line indicates the threshold at *R*_0_ = 1. The grey diamonds indicate the median *R*_0_ for bluetongue virus computed from the uncertainty analysis presented in Gubbins et al. (2012).

The posterior densities (Fig. 3) were used to calculate the basic reproduction number (*R*_0_) for SBV in cattle and sheep and its dependence on temperature (Fig. 4). For both species, *R*_0_ increases with temperature up to 21 °C, after which it decreases. Moreover, the threshold at *R*_0_ = 1 is exceeded for temperatures between 13 and 34 °C (Fig. 4). The basic reproduction number is slightly higher for sheep compared with cattle (Fig. 4), which is a consequence of the longer duration of viraemia in sheep compared with cattle (Fig. 3c and d).

Results for the analyses of the individual data-sets (Table S3; Figs. S3 and S4) were broadly consistent with those for the combined analysis (Table 3; Fig. 2, Fig. 3), though there were some small differences in the estimates.

### 3.2 Transmission of Schmallenberg virus between farms

**Fig. 5:**
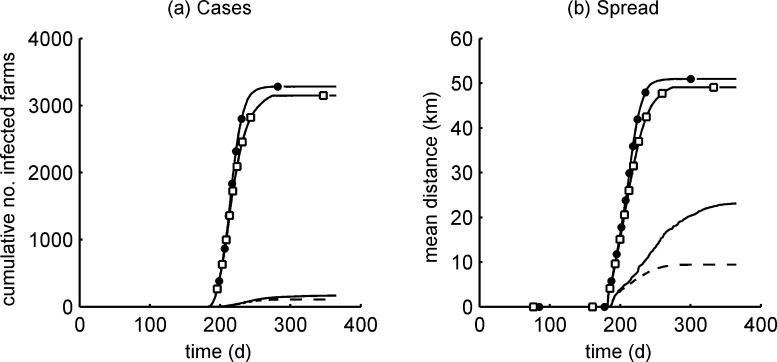
Predicted cumulative (a) number of cases and (b) spread vs. time for regional outbreaks of BTV and SBV. Each figure shows results for the regional spread of BTV with no movement restrictions (solid line), SBV with no movement restrictions (filled circle), BTV with standard movement restrictions imposed during an outbreak in the UK (dashed line) and SBV with a total movement ban (open square). Each line is the mean of 100 simulations. Infection was introduced on day 182 (i.e. 1 July).

The mean cumulative number of cases and mean cumulative spread on day 365 are given in Table 4 for each parameter set. For the cases of BTV and SBV, with and without movement restrictions, these measures are plotted against time (Fig. 5). A small reduction in the probability of transmission from vector to host (*b*) (from 0.9 [BTV] to 0.76 [SBV]) led to a negligible reduction in the cumulative number of cases and distance spread (Table 4; BTV vs. set 2). A large increase in the probability of transmission from host to vector (*β*) (from 0.01 [BTV] to 0.14 [SBV]) led to a 7.2-fold increase in the cumulative number of cases and 1.5-fold increase in the distance spread (Table 4; BTV vs. set 3). The short incubation period of SBV (2 days, compared to 5–7 for BTV) more than tripled the cumulative number of cases and increased the distance spread (Table 4; BTV vs. set 4). The short durations of SBV relative to BTV viraemia dramatically reduced the cumulative number of cases and distance spread (Table 4; BTV vs. set 5). The relationship between temperature and EIP for SBV led to a five-fold increase in the cumulative cases and 36% increase in spread relative to BTV (Table 4; BTV vs. set 6). Therefore, most parameters for SBV increase the scale and size of outbreaks (compared to BTV), while one (short viraemia) decreases them. However, the net effect of including all of the SBV parameters together is a 20-fold increase in the cumulative number of cases and doubling of the distance spread (without movement restrictions) (Table 4; BTV vs. SBV).

**Table 4:** Impact of epidemiological parameters and movement restrictions on predicted regional spread (cumulative number of affected farms and extent of spread in km) of BTV and SBV.

Parameter set	Description	Movement restrictions			
		No		Yes	
		No. farms	Radius	No. farms	Radius
BTV	All estimates for BTV	167	23.2	109	9.4
Set 2	As BTV, except probability of transmission from vector to host for SBV	149	21.7	–	–
Set 3	As BTV, except probability of transmission from host to vector for SBV	1201	34.9	–	–
Set 4	As BTV, except incubation period for SBV	536	28.0	–	–
Set 5	As BTV, except recovery rates in cattle and sheep for SBV	14	7.9	–	–
Set 6	As BTV, except EIP parameters for SBV	821	31.6	–	–
SBV	All estimates for SBV	3281	50.9	3148	49.1

The model suggests that the mean cumulative number of cases for SBV, without any movement restrictions, is thirty times greater than the number predicted for BTV with standard BTV movement restrictions (as described in Turner et al., 2012) (Fig. 5a). As targeted movement restrictions would not be possible for SBV without a widespread surveillance programme, we considered the effect of imposing a total movement ban. The model indicates that such a ban could reduce the mean cumulative number of cases of SBV by only about 4% (Fig. 5a) and the distance spread by only 3.5% (Fig. 5b). By contrast, a BTV outbreak appears to be much more sensitive to the effects of movement restrictions. Imposing UK standard movement restrictions (i.e. less stringent than a total ban) achieves a 35% reduction in cumulative cases and 60% reduction in the distance spread (Fig. 5).

## 4 Discussion

In the present study we have used approximate Bayesian computation (Marjoram et al., 2003, Toni et al., 2009, Sunnaker et al., 2013) to estimate epidemiological parameters for SBV. This allowed us to identify parameters consistent with the available data, but without the need to evaluate a complex likelihood function. Using this approach it was possible to draw inferences about the model parameters (Table 3; Fig. 3) even when only limited data are available (in our case, a single observation of within-farm seroprevalence for each farm). Indeed, we were able to obtain reasonable posterior estimates even using the smallest data-set in the study (seroprevalence in sheep in Belgium), which provides a single observation for 82 farms. However, the methods did make use of informative priors (Table 3) to help constrain the areas of parameter space explored by the methods.

In several previous studies, BTV has been used as a proxy when studying SBV (European Food Safety Authority, 2012a, European Food Safety Authority, 2012b, Bessell et al., 2013), yet our analysis of within-farm spread has highlighted three key differences between these two viruses. First, the duration of viraemia is much shorter in both cattle and sheep, typically around 3–4 days (Table 3; Fig. 3) compared with 16–20 days for BTV (see Gubbins et al., 2008 and references therein). Despite this much shorter duration of viraemia (and, hence, infectiousness) the within-farm seroprevalence for SBV (Fig. 2; see Méroc et al., 2013a, Méroc et al., 2013b, Veldhuis et al., 2013) is still typically higher than was observed at a similar point in the outbreak of BTV serotype 8 (BTV-8) in northern Europe in 2006/7 (Elbers et al., 2008, Méroc et al., 2008, van Schaik et al., 2008). This observation can be accounted for by the second and third differences between SBV and BTV: the probability of transmission from host to vector and virus replication.

The probability of transmission from host to vector was estimated to be 14% (95% credible interval (CI): 8–27%) (Table 3), which is slightly lower than that estimated for SBV in colony-reared *C. sonorensis*, a North American vector species (19%; 95% CI: 14–23%) (Veronesi et al., 2013). This compares with estimates for the probability of transmission from host to vector for BTV in field-caught *Culicoides* populations of around 1% (Carpenter et al., 2006, Carpenter et al., 2008). In the model, the posterior mean for the peak prevalence of SBV-infected midges was 0.48% (95% CI: 5 × 10^−4^ to 2.64%), which is consistent with reported prevalences in the field (De Regge et al., 2012, Elbers et al., 2013).

In terms of virus replication, SBV is predicted by the model to have a lower threshold temperature for replication (12.3 °C) and to replicate at a faster rate above the threshold (0.03 per day-degree) (Table 3) than has been reported for any strain of BTV (Carpenter et al., 2011). However, there are currently only very limited data on SBV replication in *Culicoides* biting midges, which precludes comparison with our indirect inferences from the transmission model.

Combining the posterior estimates for the individual epidemiological parameters in the basic reproduction number, *R*_0_ (see Eq. (4)), shows that, despite the short duration of viraemia, the combination of a higher probability of transmission from host to vector and faster virus replication result in high values for *R*_0_ (peak *R*_0_ is approximately 6.2 for cattle-only farms and 7.6 for sheep-only farms; Fig. 4) and exceeds the threshold at *R*_0_ = 1 for a wide range of temperatures (13–34 °C) (Fig. 4). This contrasts with estimates previously derived for BTV (Gubbins et al., 2008, Gubbins et al., 2012) for which the peak *R*_0_ is lower (3.8 in cattle and 3.4 in sheep) and for which the threshold of *R*_0_ = 1 is exceeded for a somewhat narrower range of temperatures (14–31 °C) (Fig. 4).

Incorporating the parameter estimates for SBV transmission obtained from the within-farm model in a between-farm model indicated that three characteristics of SBV (compared to BTV) increased outbreak size and spread (namely, the higher probability of transmission from host to vector, the shorter latent period and modified virus replication rate with temperature), while one decreased them (shorter duration of viraemia). The net effect, however, is that SBV is predicted to infect many times more animals, and spread considerably further, than BTV in the same time period.

Applying movement restrictions based on zones around infected premises (as is the case for BTV) is unlikely to be feasible for SBV, because the detection of infected farms would require extensive active surveillance. In this case, a total movement ban might be a more straightforward approach, though our model shows that even a total movement ban is expected to have only very minor effect on the final size and spread of an SBV outbreak.

A limitation of our simulations of the between-farm spread of SBV is that they were run for a single region of the UK. Farming in this region is not atypical of the rest of the UK, with significant numbers of both cattle, sheep and mixed farms. It is a relatively homogenous environment, however, compared to many other parts of the UK and mainland Europe. Heterogeneity in host and vector density can act to reduce the scale of vector-borne disease outbreaks (see Charron et al., 2013 for the case of BTV), perhaps because pockets of low vector or host density impede spread of the infection. Equally, the UK region in which transmission was modelled lacks hills and mountains, which might present further barriers to the spread of SBV. Our model did not assume homogeneity of vector or host densities (host densities were based on reported animal numbers, and vector densities were assumed to scale with them, and so are equally heterogeneous). However, it remains possible that simulations of spread in other regions of the UK or mainland Europe, with more heterogeneous patterns of farming and geography would generate outbreaks which spread more slowly and which are of a smaller final size.

While simulated outbreaks in other regions might be smaller than those presented here, the relative importance of animal and vector movements is unlikely to be radically altered. Indeed, a recent study of the SBV outbreak in continental Europe in 2011 concluded that SBV spread in the region can be largely explained by vector movements on the wind (Sedda and Rogers, 2013). Furthermore, the UK has extensive animal movements, yet we find that stopping them does very little to slow the spread of SBV. In other countries, with less extensive animal movements, it is even less likely that stopping these movements will help prevent the spread of the disease.

## 5 Conclusions

Changes to four epidemiological parameters (latent period, duration of viraemia, probability of transmission from host to vector and virus replication) are sufficient to account for the differences in the transmission of SBV within and between farms when compared with BTV-8. This suggests that alternative transmission mechanisms (e.g. direct transmission, additional vector species) (Garigliany et al., 2012, Davies and Daly, 2013) are not necessary to explain the observed patterns of spread of SBV, though they may still play a minor role. This is consistent with the results of challenge studies which have been published to date (Wernike et al., 2013). The enhanced between-farm transmission of SBV, relative to BTV, brought about by these four changes is such that the application of movement restrictions, even a total animal movement ban, has little effect on the final outcome.

## Conflict of interest

None declared.
