# Supplementary material for: Inferences about the transmission of Schmallenberg virus within and between farms
Source: Prev Vet Med. 2014 Oct 15;116(4):380–90. doi: 10.1016/j.prevetmed.2014.04.011 (PMC4204990; doi:10.1016/j.prevetmed.2014.04.011)
Supplement: Supplementary file 1 [file mmc1.docx]

***Electronic supplementary material Preventive Veterinary Medicine***

**Inferences about the transmission of Schmallenberg virus within and between farms**

Simon Gubbins^1,*^, Joanne Turner^2^, Matthew Baylis^2^, Yves van der Stede^3,4^, Gerdien van Schaik^5^, José Cortiñas Abrahantes^6^ & Anthony J. Wilson^1^

^1^ *The Pirbright Institute, Ash Road, Pirbright, Surrey GU24 0NF, U.K.*

^2^ *Department of Epidemiology and Population Health, Institute of Infection and Global Health, University of Liverpool, Leahurst Campus, Chester High Road, Neston, Cheshire CH64 7TE, U.K.*

^3^ *Unit of Co-ordination Veterinary Diagnosis-Epidemiology and Risk Assessment, CODA-CERVA, Groeselenberg 99, 1180 Brussels, Belgium*

^4^ *Department of Virology, Parasitology and Immunology, Faculty of Veterinary Medicine, Ghent University, Salisburylaan 133, 9820 Merelbeke, Belgium*

^5^ *GD Animal Health, Arnsbergstraat 7, 7413EZ Deventer, The Netherlands*

^6^ *European Food Safety Authority, Via Carlo Magno 1A, 43126 Parma, Italy*

^*^ Corresponding author:

email: [simon.gubbins@pirbright.ac.uk](mailto:simon.gubbins@pirbright.ac.uk); Tel. +44 1483 232441; Fax +44 1483 232448

**S1 Derivation of within-farm prevalence curves for the between-farm transmission model**

The model for the transmission of Schmallenberg virus (SBV) between farms (see section 2.2 in the main paper) uses the prevalence curve described by Pongsumpun et al. (2008) to approximate the within-farm transmission dynamics. In this case, the prevalence of infection *t* days after the farm became infectious is given by,

$$p(t)=\frac{p_{\lim}}{4}\left[1-\tanh\left(\frac{L_{1}-t}{T_{1}}\right)\right]\left[1-\tanh\left(\frac{t-L_{2}}{T_{2}}\right)\right],$$

where the parameters *p*_lim_, *L*_1_, *L*_2_, *T*_1_ and *T*_2_ do not have a biological meaning, but are selected to achieve a suitable shape for the prevalence curve. Parameters for the within-farm prevalence curve, , were computed by fitting it to an ordinary differential equation (ODE) model which is related to the stochastic within-farm model (see section 2.1 in the main paper) by incorporating similar transition rates from one class to another and the same parameter values.

More precisely, the ODE model describes the change over time in the proportion of cattle and sheep which are susceptible (*w_i_*), infected, but not yet infectious (*x_i_*), infectious (*y_i_*) and recovered (*z_i_*) (where *i* indicates cattle (*C*) or sheep (*S*)). It also describes the change over time in the proportion of vectors on the farm which are susceptible (*S*), infected, but not yet infectious (*L*) and infectious (*I*). The dynamics of SBV infection in the cattle or sheep populations are described by the following set of linked ODEs,

$$\begin{matrix} \frac{\text{d}w_{i}}{\text{d}t}=-\lambda_{i}w_{i}, \\ \frac{\text{d}x_{i}}{\text{d}t}=\lambda_{i}w_{i}-c_{i}y_{i}, \\ \frac{\text{d}y_{i}}{\text{d}t}=c_{i}x_{i}-r_{i}y_{i}, \\ \frac{\text{d}z_{i}}{\text{d}t}=r_{i}y_{i}, \end{matrix}$$

while those in the vector population are described by,

$$\begin{matrix} \frac{\text{d}S}{\text{d}t}=\mu-\lambda_{V}S-\mu S, \\ \frac{\text{d}L}{\text{d}t}=\lambda_{V}S-(\nu+\mu)L, \\ \frac{\text{d}I}{\text{d}t}=\nu L-\mu I, \end{matrix}$$

where,

are the forces of infection for cattle and sheep, respectively (cf. equation (1)), ϕ is the proportion of bites that fall on cattle (calculated as ϕ=*H_C_*/(*H_C_*+σ*H_S_*))) and,

$$\lambda_{V}=\beta a(y_{C}+y_{S}),$$

is the force of infection for vectors (cf. equation (3)). Importantly, all the parameters in the ODE model given by equations - are the same as defined in Table 2 in the main paper (i.e. for the stochastic within-farm model), except for 1/*c_i_* which is the mean latent period for SBV (assumed to be 2 days for both cattle and sheep; Hoffmann et al. 2012).

The ODE model was solved numerically to produce a combined host prevalence curve (i.e. *p*_ODE_(*t*)=*y_C_*(*t*)+*y_S_*(*t*)) for each of seven sets of parameter values (Tables S1 & S2), fixing the temperature-dependent parameters at their values at 20 °C. Parameters for the prevalence curve, , were then computed by fitting it to the prevalence curve generated by the ODE model using a nonlinear least-squares method with bisquare weighting of the residuals.

The prevalence curve, , (including the fitted values for parameters *p*_lim_, *L*_1_, *L*_2_, *T*_1_ and *T*_2_) was used in the between-farm model to approximate the within-farm dynamics during a period of vector activity (labelled ‘summer’ in Fig. S1). For periods of vector inactivity (labelled ‘winter’ in Fig. S1), an exponential curve was used with a decay rate that ensured that the ‘winter’ curve fell below the corresponding ‘summer’ curve (i.e. vector activity is curtailed by winter). Prevalence curves for bluetongue virus (BTV) are also shown, to demonstrate visually the different within-farm dynamics used for the two viruses.

Finally, as the latent period for SBV is much shorter than for BTV, the cumulative probability that a farm changes SBV status from exposed to infected was set to increase much more rapidly (Figure S2) than is the case with BTV in Turner et al. (2012).


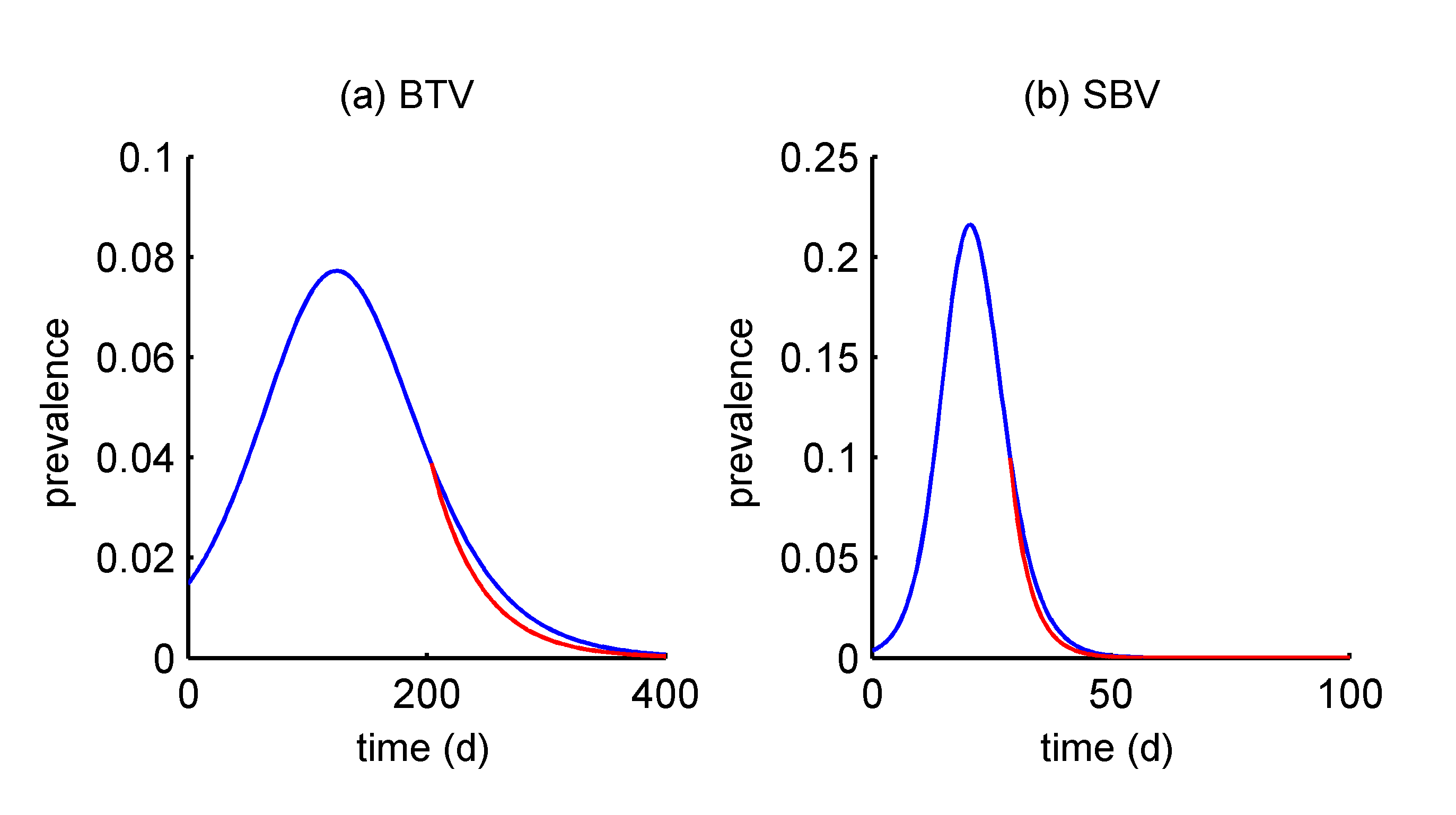


**Figure S1**. (*a*,*b*) Prevalence curves used to approximate within-farm dynamics in the between-farm model for the transmission of (*a*) BTV and (*b*) SBV between farms. The curves are those when vectors are active (‘summer’; blue lines) or when they are inactive (‘winter’; red lines). Time is ‘time since farm became infectious’. Prevalence indicates degree of infectivity and hence risk to other farms via vector and animal movements.


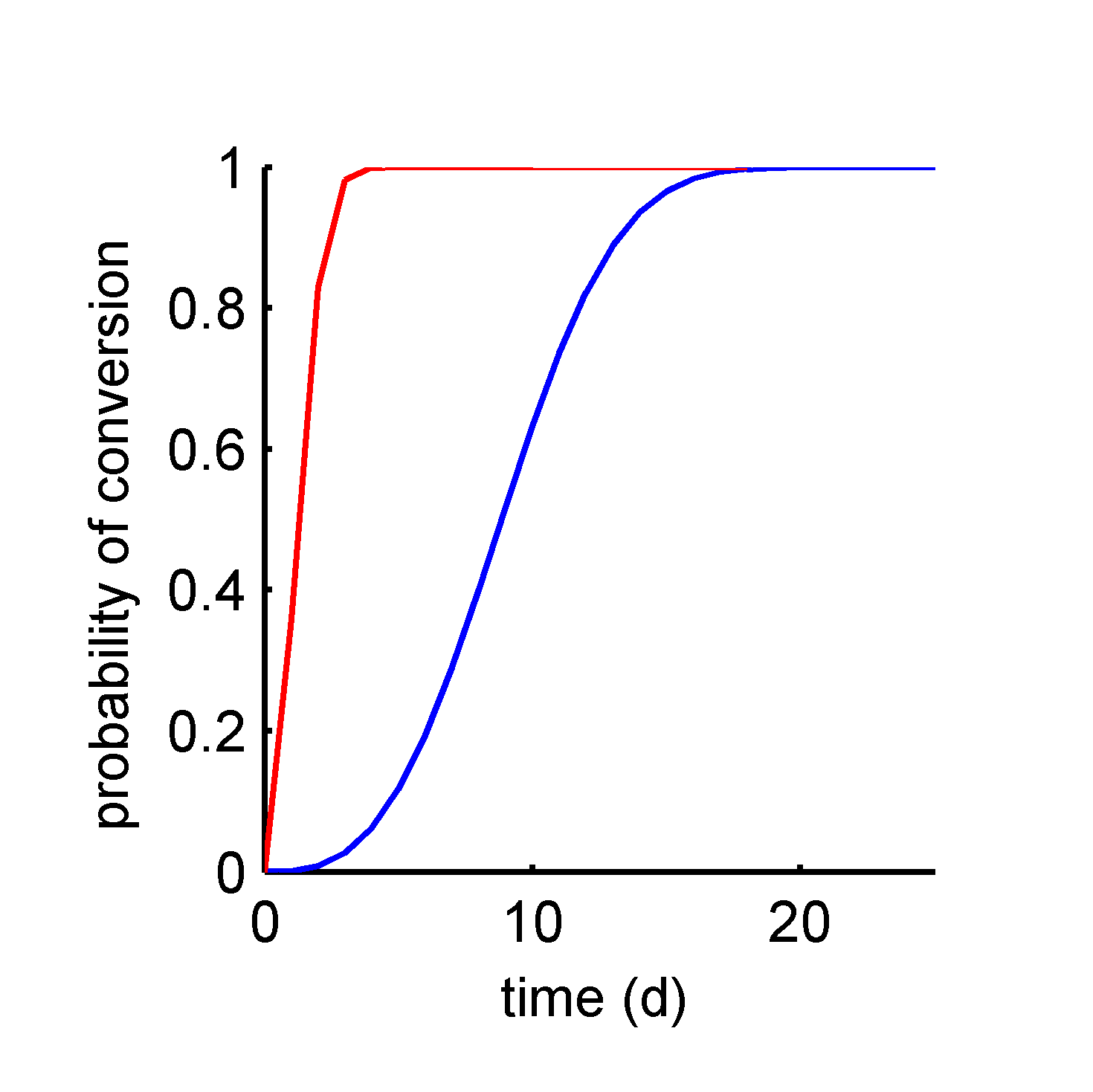


**Figure S2**. Curves representing cumulative probability that a farm changes status from exposed (E) to infected (I) for BTV (blue line) or SBV (red line). Time is ‘time since farm became exposed’.

**Table S1.** Parameter sets used in the between-farm model to explore the sensitivity of model predictions to differences in parameters between BTV and SBV. Values in bold are the posterior median values for SBV taken from Table 3. Parameter values for SBV replaced those for BTV as follows: estimated vector to host transmission rate (set 2); estimated host to vector transmission rate (set 3); short, 2 day, incubation period for SBV (set 4); estimated recovery rate in cattle and sheep (set 5); estimated relationship between temperature and EIP (set 6).

| parameter | symbol | parameter set (identifier as used in main paper) | | | | | | |
| --- | --- | --- | --- | --- | --- | --- | --- | --- |
|  |  | BTV | set 2 | set 3 | set 4 | set 5 | set 6 | SBV |
| host incubation rate (cattle) | *c*_C_ | 1/7 | 1/7 | 1/7 | **1/2** | 1/7 | 1/7 | **1/2** |
| host incubation rate (sheep) | *c*_S_ | 1/5 | 1/5 | 1/5 | **1/2** | 1/5 | 1/5 | **1/2** |
| recovery rate (cattle) | *r*_C_ | 1/20.6 | 1/20.6 | 1/20.6 | 1/20.6 | **1/3.04** | 1/20.6 | **1/3.04** |
| recovery rate (sheep) | *r*_S_ | 1/16.4 | 1/16.4 | 1/16.4 | 1/16.4 | **1/4.37** | 1/16.4 | **1/4.37** |
| probability of transmission from vector to host | *b* | 0.9 | **0.76** | 0.9 | 0.9 | 0.9 | 0.9 | **0.76** |
| probability of transmission from host to vector | *β* | 0.01 | 0.01 | **0.14** | 0.01 | 0.01 | 0.01 | **0.14** |
| rate at which exposed vectors become infectious | ν(*T*) |  |  |  |  |  |  |  |
| ν(*T*)=max(0,0.0003*T*(*T*-10.4)) |  | Y | Y | Y | Y | Y | **N** | **N** |
| ν(*T*)=max(0,0.03(*T*-12.35) |  | N | N | N | N | N | **Y** | **Y** |
| ‘peak’ prevalence taking into account current susceptibility | *p*_lim_ | 0.19 | 0.16 | 1 | 0.24 | 0.02 | 0.73 | 0.44 |
| parameter of prevalence curve | *L*_1_ | 96.5 | 123.8 | 32.6 | 83.1 | 7.5 | 48.9 | 16.9 |
| parameter of prevalence curve | *L*_2_ | 147.3 | 155.2 | 43.9 | 115.7 | 7.5 | 60.3 | 23.5 |
| parameter of prevalence curve | *T*_1_ | 80.2 | 112.5 | 10.4 | 66.7 | 3.9 | 27.6 | 7.03 |
| parameter of prevalence curve | *T*_2_ | 90.7 | 119.3 | 29.4 | 82.6 | 30.2 | 42.6 | 9.03 |
| rate of decline in vector-free period | γ | 0.02 | 0.02 | 0.08 | 0.03 | 0.07 | 0.06 | 0.24 |
| parameter of E to I curve | *λ*_C_ | 10 | 10 | 10 | **1.5** | 10 | 10 | **1.5** |
| parameter of E to I curve | *k*_C_ | 3 | 3 | 3 | **2** | 3 | 3 | **2** |

**Table S2.** Parameters of the between-farm model that remain fixed at values used in the original BTV model (Turner et al. 2012).

| parameter | symbol | value |
| --- | --- | --- |
| temperature (fixed for curve fitting only) | *T* | 20 |
| reciprocal of the time interval between blood meals | *a* | see Table 2 |
| vector to host ratio (cattle) | *m*_C_ | 500 |
| vector to host ratio (sheep) | *m*_S_ | 500 |
| vector feeding preference | σ | 0.5 |
| proportion of bites that fall on cattle | ϕ | *H_C_*/(*H_C_*+σ*H_S_*) |
| vector mortality rate | μ | see Table 2 |
| vector replacement rate | ρ | see Table 2 |
| number of hosts (cattle) | *H*_C_ | 100 |
| number of hosts (sheep) | *H*_S_ | 100 |

**S2 Parameter estimation using individual seroprevalence data-sets**

In addition to fitting the model for the within-farm transmission of SBV to all four seroprevalence data-sets in a single analysis, we also fitted the model to each data-set independently. The results are broadly consistent across the data-sets (Figs S3 & S4; Table S3; cf. Figs 1 & 2; Table 1 in the main paper), though there are some small differences. However, the fit to the individual data-sets was somewhat better than when fitting to all four data-sets simultaneously (Fig. S3; cf. Fig. 1).


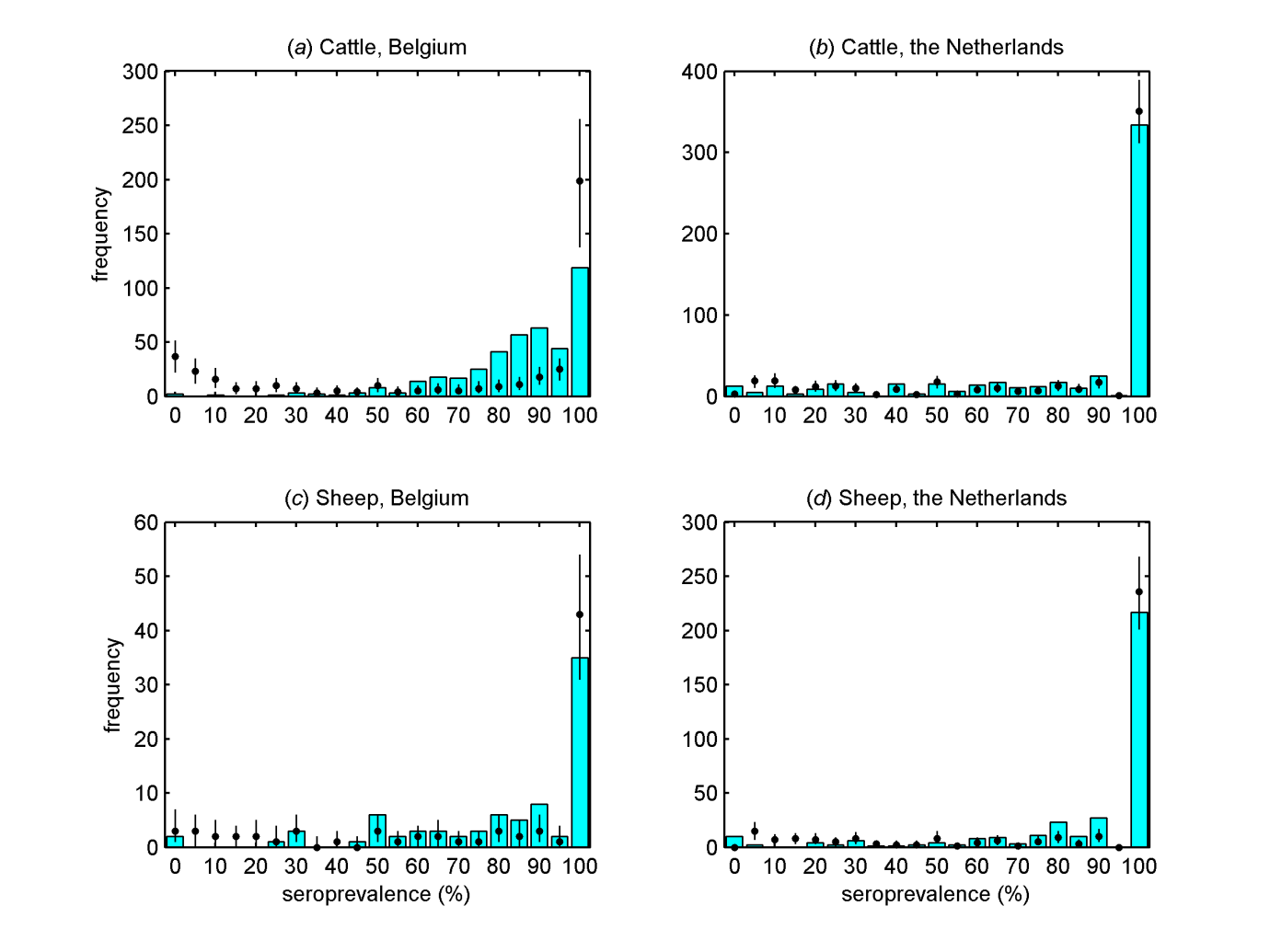


**Fig. S3.** Observed (bars) and expected (median (symbols) and 95% prediction intervals (error bars)) distribution of within-farm seroprevalence in (*a*,*b*) cattle and (*c*,*d*) sheep from (*a*,*c*) Belgium and (*b*,*d*) The Netherlands. Results in each figure are for when the model for the within-farm transmission of SBV was fitted to each data-set independently.


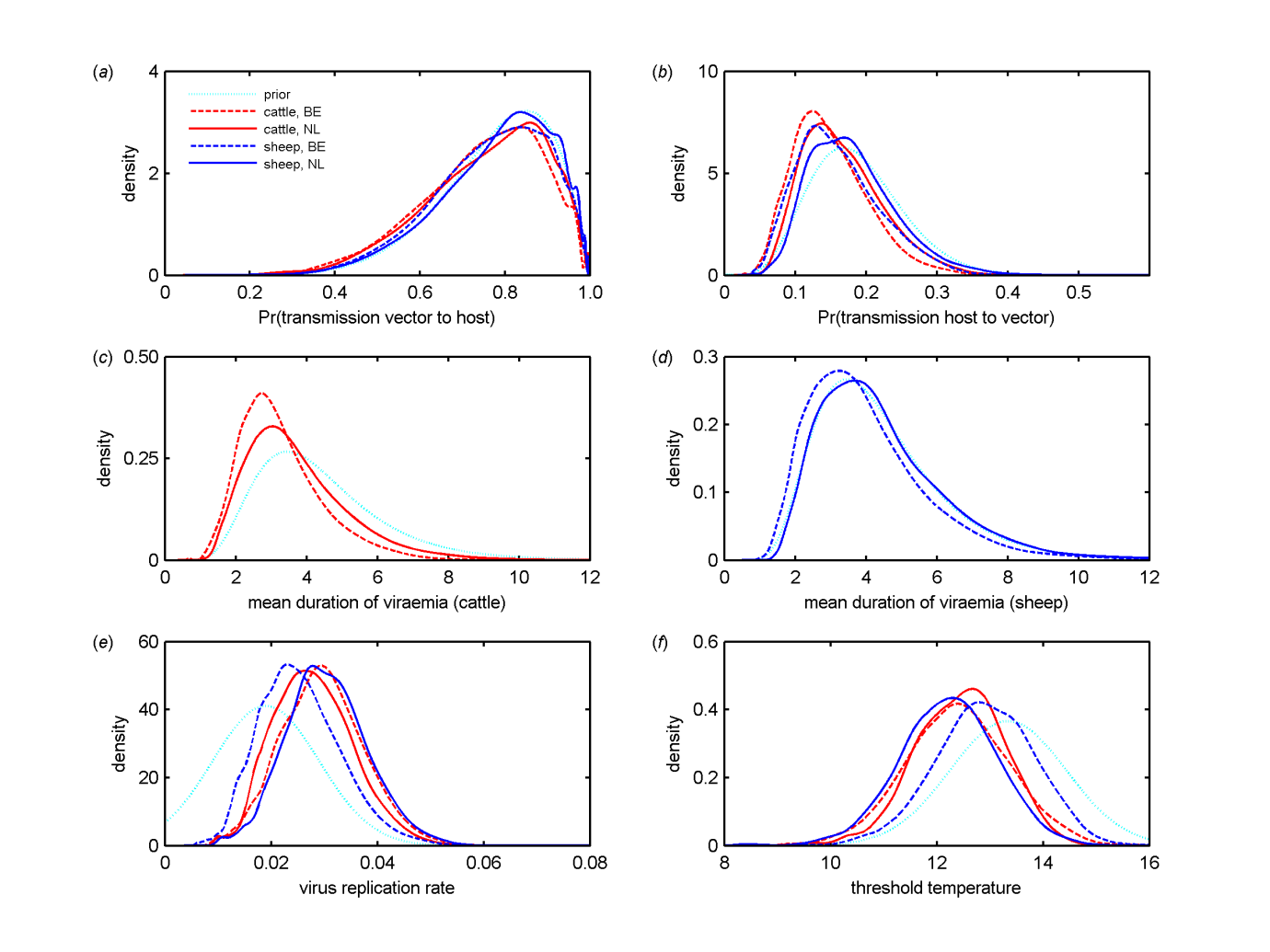


**Fig. S4.** Posterior distributions for epidemiological parameters for SBV: (*a*) probability of transmission from vector to host; (*b*) probability of transmission from host to vector; (*c,d*) mean duration of viraemia (days) in (*c*) cattle or (*d*) sheep; (*e*) virus replication rate; and (*f*) threshold temperature (°C) for virus replication. Each figure shows the prior (dotted cyan line; see Table 2 in the main paper) and the posterior when the model for the within-farm transmission of SBV was fitted independently to data on within-farm seroprevalence in cattle in Belgium (BE) (dashed red line), cattle in The Netherlands (NL) (solid red line), sheep in Belgium (dashed blue line), or sheep in The Netherlands (dashed blue line).

**Table S3.** Posterior median and 95% credible intervals (CI) for parameters in the model for the within-farm transmission of SBV when fitting to seroprevalence data-sets for individual species and countries.

| Parameter | Cattle, Belgium | | Cattle, The Netherlands | | Sheep, Belgium | | Sheep, The Netherlands | |
| --- | --- | --- | --- | --- | --- | --- | --- | --- |
|  | median | 95% CI | median | 95% CI | median | 95% CI | median | 95% CI |
| *probability of transmission* | | |  |  |  |  |  |  |
| vector to host | 0.76 | (0.43, 0.96) | 0.78 | (0.45, 0.96) | 0.78 | (0.47, 0.96) | 0.80 | (0.48, 0.97) |
| host to vector | 0.14 | (0.07, 0.27) | 0.16 | (0.08, 0.29) | 0.15 | (0.07, 0.29) | 0.17 | (0.09, 0.32) |
| *duration of viraemia (cattle)* | |  |  |  |  |  |  |  |
| mean (days) | 3.05 | (1.57, 6.14) | 3.46 | (1.70, 7.43) | - | - | - | - |
| no. stages | 12 | (2, 20) | 12 | (2, 20) | - | - | - | - |
| *duration of viraemia (sheep)* | |  |  |  |  |  |  |  |
| mean (days) | - | - | - | - | 3.72 | (1.72, 8.41) | 4.09 | (2.02, 8.56) |
| no. stages | - | - | - | - | 11 | (2, 20) | 12 | (2, 20) |
| *extrinsic incubation period* | |  |  |  |  |  |  |  |
| virus replication rate | 0.030 | (0.015, 0.045) | 0.028 | (0.016, 0.043) | 0.024 | (0.012, 0.041) | 0.030 | (0.017, 0.046) |
| threshold temperature | 12.38 | (10.41, 14.28) | 12.48 | (10.77, 13.95) | 12.91 | (11.02, 14.57) | 12.21 | (10.40, 13.89) |
| no. stages | 6 | (2, 37) | 8 | (2, 41) | 8 | (2, 37) | 7 | (2, 31) |

**References**

Gubbins, S., Carpenter, S., Baylis, M., Wood, J.L.N., Mellor, P.S., 2008. Assessing the risk of bluetongue to UK livestock: uncertainty and sensitivity analysis of a temperature-dependent model for the basic reproduction number. J. R. Soc. Interface 5, 363-371.

Hoffmann, B., Scheuch, M., Höper, D., Jungblut, R., Holsteg, M., Schirrmeier, H., Eschbaumer, M., Goller, K.V., Wernike, K., Mettenleiter, T.C., Beer, M., 2012. Novel orthobunyavirus in cattle, Europe, 2012. Emerg. Infect. Dis. 18, 469-472.

Pongsumpun, P., Garcia Lopez, D., Favier, C., Torres, L., Llosa, J., Dubois, M.A., 2008. Dynamics of dengue epidemics in urban contexts. Trop. Med. Intl Health 13, 1180-1187.

Turner, J., Bowers, R.G., Baylis, M., 2012. Modelling bluetongue virus transmission between farms using animal and vector movements. Sci. Reports 2, 319.
